# Classical *ctxB* in *Vibrio cholerae* O1, Kolkata, India

**DOI:** 10.3201/eid1501.080543

**Published:** 2009-01

**Authors:** Amit Raychoudhuri, Tapas Patra, Kausik Ghosh, Thandavarayan Ramamurthy, Ranjan K. Nandy, Yoshifumi Takeda, G. Balakrish Nair, Asish K. Mukhopadhyay

**Affiliations:** National Institute of Cholera and Enteric Diseases, Kolkata, India (A. Raychoudhuri, T. Patra, K. Ghosh, T. Ramamurthy, R.K. Nandy, G.B. Nair, A.K. Mukhopadhyay); Collaborative Research Center of Okayama University for Infectious Diseases in India, Kolkata (Y. Takeda)

**Keywords:** Vibrio cholerae, ctxB, El Tor, classical, letter

**To the Editor:** Among the 206 serogroups of *Vibrio cholerae,* O1 and O139 are associated with epidemic cholera. Serogroup O1 is classified into 2 biotypes, classical and El Tor. Conventionally, the 2 biotypes can be differentiated on the basis of a set of phenotypic traits. Comparative genomic analysis has shown variations in different genes between these biotypes (*1*). Cholera toxin (CT), the major toxin responsible for the disease cholera, has 2 epitypes or immunologic forms, CT1 and CT2 (*2*). Another classification recognizes 3 genotypes on the basis of the *ctxB* gene sequence variation (*3*). In the past few years, a new emerging form of *V. cholerae* O1, which possesses traits of both classical and El Tor biotypes, has been isolated in Bangladesh (*4**,**5*), Mozambique (*6*), Vietnam, Hong Kong, Japan, and Zambia (*7*). These strains were variously labeled as Matlab variants, hybrids, or altered El Tor strains.

Our study analyzed, in chronological order, strains of *V. cholerae* O1 that were isolated over 17 years (1989–2005). We used strains isolated during diarrhea surveillance conducted at the Infectious Diseases Hospital, Kolkata (Calcutta), to determine precisely when the hybrid strains appeared in this region. A total of 171 strains of *V. cholerae* O1, which were selected to cover different months of each year, were included in this study, along with 2 reference strains for classical and El Tor biotypes. The *V. cholerae* strains were confirmed serologically by slide agglutination using a specific polyvalent antiserum to *V. cholerae* O1.

We focused on the *ctxB* gene. The strains were examined by mismatch amplification mutation assay (MAMA)–based PCR for detecting the *ctxB* allele; a common forward primer was used for 2 alleles, FW-Com (5′-ACTATCTTCAGCATATGCACATGG-3′); and 2 allele-specific primers, Re-cla (5′-cctggtacttctacttgaaacg-3′) and Re-elt (5′-CCTGGTACTTCTACTTGAAACA-3′), were used for classical and El Tor biotypes, respectively (*8*). Results of the MAMA-PCR are summarized in the Table. All of the 123 *V. cholerae* O1 strains from 1995 through 2005 yielded only the classical type of *ctxB*, which indicates that since 1995 the classical type has completely replaced the El Tor type *ctxB* (Table). To reconfirm our PCR-based results, we selected 25 representative strains for DNA sequencing of the *ctxB* gene. The deduced amino acid sequences were aligned with the CtxB sequences of reference strains N16961 (El Tor) and O395 (classical). The deduced amino acid sequences of all 25 strains were identical to those of the classical reference strain; histidine was at position 39 and threonine was at position 68. Thus, the results from DNA sequencing of the *ctxB* gene confirmed the MAMA-PCR results.

**Table:** Prevalence of different types of *ctxB* alleles among *Vibrio cholerae* O1 strains, Kolkata, India, 1989–2005

Year isolated	No. strains tested	No. alleles		
		Classical *ctxB*	El Tor *ctxB*	Classical + El Tor *ctxB*
1989	6	0	6	0
1990	7	4	3	0
1991	10	8	0	2*
1992	10	4	5	1*
1993	6	4	2	0
1994	9	8	1	0
1995	23	23	0	0
1996	10	10	0	0
1997	10	10	0	0
1998	10	10	0	0
1999	10	10	0	0
2000	10	10	0	0
2001	10	10	0	0
2002	10	10	0	0
2003	10	10	0	0
2004	10	10	0	0
2005	10	10	0	0

*These strains carry the *ctxB* gene for El Tor as well as classical strains.

Our results highlight a noteworthy event in the evolution of recent *V. cholerae* strains. Analysis of type *ctxB* that had been circulating in Kolkata for 17 years (1989–2005) showed that in 1989 only the El Tor allele of *ctxB* was present. Our results further indicate that classical type *ctxB* emerged in 1990, although El Tor type *ctxB* was still present in almost equal numbers during that year. During 1991, a unique event took place when the classical type became predominant, along with strains having both classical and El Tor type *ctxB*. In 1994, isolation of strains with El Tor *ctxB* became rare, and the major *ctxB* allele was of the classical type. *V. cholerae* O1 strains from 1995 onward were found to carry classical type *ctxB*, which totally replaced the El Tor type *ctxB* allele.

Replacement of El Tor type *ctxB* by the classical allele has been reported in Bangladesh since 2001 (*5*), but this event seems to have occurred earlier in Kolkata. Perhaps the new type of El Tor strains arose when *V. cholerae* strains with typical seventh pandemic El Tor genetic background were replaced with strains having the *ctxB* gene, possibly driven by selective pressure to survive and adapt better in host intestines. Considering the increase in the global prevalence of cholera (*9*), the origin and spread of these new variants of *V. cholerae* strains should be tracked in the population by genome analysis. Finally, this study has described a brief period from February 1991 through December 1992 when El Tor strains had CTX prophages of both classical and El Tor types (data not shown), along with the *ctxB* of both biotypes. Notably, this period coincided with an unprecedented event in the history of cholera—the genesis of the O139 serogroup. After this serogroup reemerged in 1996, it harbored 2 types of CTX prophages, namely, El Tor and Calcutta (*10*). Furthermore, these strains with *ctxB* of both biotypes might also have had a pivotal role behind the emergence of El Tor strains with classical *ctxB*. Further studies are warranted to determine whether any distinct relationship exists between these overlapping events.

## References

[1] Dziejman M, Balon E, Byod D, Fraser CM, Heidelberg JF, Mekalanos JJ. Comparative genomic analysis of *Vibrio cholerae* genes that correlate with cholera endemic and pandemic diseases. Proc Natl Acad Sci U S A. 2002;99:1556–61. 10.1073/pnas.042667999 11818571 PMC122229

[2] Finkelstein RA, Burks F, Zupan A, Dallas WS, Jacob CO, Ludwig DS. Epitopes of the cholera family of enterotoxins. Rev Infect Dis. 1987;9:544–61. 2440089 10.1093/clinids/9.3.544

[3] Olsvik O, Wahlberg J, Petterson B, Uhlén M, Popovic T, Wachsmuth IK, Use of automated sequencing of polymerase chain reaction-generated amplicons to identify three types of cholera toxin subunit B in *Vibrio cholerae* O1 strains. J Clin Microbiol. 1993;31:22–5. 7678018 10.1128/jcm.31.1.22-25.1993 PMC262614

[4] Nair GB, Faruque SM, Bhuiyan NA, Kamruzzaman M, Siddique AK, Sack DA. New variants of *Vibrio cholerae* O1 biotype El Tor with attributes of the classical biotype from hospitalized patients with acute diarrhea in Bangladesh. J Clin Microbiol. 2002;40:3296–9. 10.1128/JCM.40.9.3296-3299.2002 12202569 PMC130785

[5] Nair GB, Qadri F, Holmgren J, Svennerholm AM, Safa A, Bhuiyan NA, Cholera due to altered El Tor strains of *Vibrio cholerae* O1 in Bangladesh. J Clin Microbiol. 2006;44:4211–3. 10.1128/JCM.01304-06 16957040 PMC1698305

[6] Ansaruzzaman M, Bhuiyan NA, Nair GB, Sack DA, Lucas M, Deen JL, Cholera in Mozambique, variant of *Vibrio cholerae.* Emerg Infect Dis. 2004;10:2057–9. 16010751 10.3201/eid1011.040682 PMC3329043

[7] Safa A, Sultana J, Cam PD, Mwansa JC, Kong RYC. Classical cholera toxin producing *Vibrio cholerae* O1 hybrid El Tor strains in Asia and Africa. Emerg Infect Dis. 2008;14:987–8. 10.3201/eid1406.080129 18507925 PMC2600311

[8] Morita M, Ohnishi M, Bhuiyan NA, Nusrin S, Alam M, Siddique AK, Development and validation of a mismatch amplification mutation assay PCR to distinguish between the cholera toxin B subunit of classical and El Tor biotypes of *Vibrio cholerae* O1. Microbiol Immunol. 2008;52:314–7. 10.1111/j.1348-0421.2008.00041.x 18577166

[9] World Health Organization. Cholera, 2006. Wkly Epidemiol Rec. 2007;82:273–84. 17679181

[10] Kimsey HH, Nair GB, Ghosh A, Walder MK. Diverse CTXφs and evolution of new pathogenic *Vibrio cholerae.* Lancet. 1998;352:457–8. 10.1016/S0140-6736(05)79193-5 9708764

